# Effect of the probiotic *Lactobacillus plantarum* IS-10506 on BDNF and 5HT stimulation: role of intestinal microbiota on the gut-brain axis

**Published:** 2019-04

**Authors:** Reza Ranuh, Alpha Fardah Athiyyah, Andy Darma, Vitria Prasetyo Risky, Wibi Riawan, Ingrid S. Surono, Subijanto Marto Sudarmo

**Affiliations:** 1 Department of Child Health, Dr. Soetomo Hospital, Faculty of Medicine Universitas Airlangga, Surabaya, Indonesia; 2 Department of Biomolecular Laboratory, Faculty of Medicine, Universitas Brawijaya, Malang, Indonesia; 3 Department of Food Technology, Faculty of Engineering, Bina Nusantara University, Jakarta, Indonesia

**Keywords:** Gut–brain axis, Brain-derived neurotrophic factor, Serotonin, Probiotic *Lactobacillus plantarum* IS 10506

## Abstract

**Background and Objectives:**

Microbial communities residing in the gut play a major role in the communication between the gut and the brain through neural, immune, and hormonal routes. Changes in abundance of beneficial intestinal bacteria can affect health of individuals. Conversely, drugs, disease, diet and other factors can alter the gut microbiome. However, there is limited information on the effect of exogenous factors on gut microbiota. In this study, we investigated whether a beneficial bacterium, the probiotic *Lactobacillus plantarum* IS-10506, can stimulate the gut–brain axis using Wistar rats.

**Materials and Methods:**

The animals were divided into two groups: one received *L. plantarum* IS strain 10506 supplementation, while the control group received no treatment. Activation of the gut–brain axis was evaluated by immunohistochemical analysis of intestinal and brain serotonin (5-HT) and brain neurotrophin (NT), serotonin transporter (5-HTT), and brain-derived neurotrophic factor (BDNF) levels.

**Results:**

The results showed that BDNF (*p*< 0.000), NT (*p*< 0.000007), and 5-HTT (*p*< 0.000007) expression was upregulated in the brain along with intestinal 5-HT (*p*< 0.000) level in rats treated with *L. plantarum* strain IS-10506 relative to the control group.

**Conclusion:**

The probiotic *L. plantarum* IS-10506 stimulates the gut–brain axis and can potentially promote brain development and function.

## INTRODUCTION

The digestive tract and brain are intimately connected (1). Microbial communities residing in the gut play a major role in the communication between the gut and the brain (1–3). In humans, the 200–600 million neurons of the enteric nervous system respond to chemical and mechanical stimuli from the gastrointestinal tract and combine this information with that transmitted by the brain via the vagus nerve (i.e., cranial nerve X) (4, 5). Signals between these two organs are bidirectional but most (90%) are retrograde and keep the brain informed of gut activity. The communication between the brain's emotional and cognitive centres and peripheral intestinal function is known as the gut–brain axis (1, 2, 6, 7).

Data from mice and humans have shown that gut microbiota influence the gut–brain axis; disruption of gut microbiota communities alters the expression of the neuromodulator brain-derived neurotrophic factor (BDNF), the growth factor neurotrophin (NT), and the serotonin (5-HT) transporter (5-HTT) in the hippocampus and amygdala (8–10). Additionally, clinical studies have reported that changes in gut microbiota lead to observable changes in mood or behaviour; conversely, ingestion of probiotics can positively affect brain function in healthy individuals (6, 11–13).

In the present study, we investigated whether the probiotic bacterium *Lactobaccillus plantarum* strain IS-10506, which is prevalent in Indonesia and is a typical resident of the intestine, can influence the gut–brain axis.

## MATERIALS AND METHODS

### Animals.

Male Wistar rats (8–12 weeks old; 100–120 g) were divided into two groups (n = 10 each): animals in the first served as untreated controls, and the second group received probiotic supplementation (*L. plantarum* IS-10506) for 7 days at 2.67 × 10^9^ CFU/day in 0.25 ml saline. At the end of the experiment, rats were sacrificed by cervical dislocation and the brain and small intestine (ileum) were immediately dissected. The intestinal contents were removed, and the tissues were washed with phosphate-buffered saline (PBS) and prepared for immunohistochemistry. This study was approved by the Animal Care and Use Committee of Ethics Committee of the Veterinary Faculty of Universitas Airlangga, Surabaya, Indonesia.

### Probiotic supplementation.

Microencapsulated *L. plantarum* strain IS-10506 (GenBank accession no. DQ860148) was packed in an aluminium foil sachet at the Pharmacy Installation of Dr. Soetomo Hospital (Surabaya, Indonesia) and dissolved in 1.5 ml sterile water, and administered to rats via a gastric tube once daily for 7 days at a dose of 2.67 × 10^9^ CFU/day. Probiotic viability was assessed 1 week prior to the treatment.

### Immunohistochemistry.

Ileum and brain tissues were fixed in 10% formalin solution, followed by dehydration and paraffin embedding. Serial sections of the tissues were done by cleaned and fixed in 1n 10% formallin buffer solution. Then, this procedure followed by dehydration, clearing, and embedding. Tissue sections were probed with antibodies against 5-HT (YC5/45 sc-58031, Santacruz Biotech, USA), BDNF ((n20) sc-546, Santacruz Biotech, USA), 5-HTT (SR2A antibody (A-4) sc-166775, Santacruz Biotech, USA) and NT (sc-365444, Santacruz Biotech, USA). The sections were observed under a light microscope (CX21; Olympus, Tokyo, Japan) and photographed with an ILCE6000 camera (Sony, Tokyo, Japan). The number of immunopositive cells in 20 random fields at 1000× magnification was counted.

### Statistical analysis.

Differences between groups were evaluated with a paired sample t test and one-way analysis of variance for normally distributed data. Significance was set at *p*< 0.05.

## RESULTS

Rats that received *L. plantarum* IS-10506 supplementation showed an increased number of 5-HT-positive cells in the ileum relative to the control group ( *p*< 0.0001; Figs. 1–3). Similar trends were observed in the brain levels of BDNF ( *p*< 0.0001; Figs. 4–6), 5-HTT ( *p*< 0.000007; Figs. 7–9), and NT ( *p*< 0.000007; Figs. 10–12): in each case, the number of immunopositive cells was higher in the treatment group than in control rats.

**Fig. 1.**
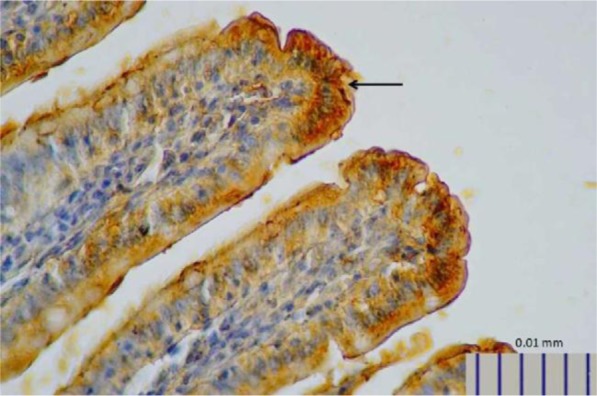
Representative image of intestinal 5-HT expression in control rats, as detected by immunohistochemistry (400× magnification).

**Fig. 2.**
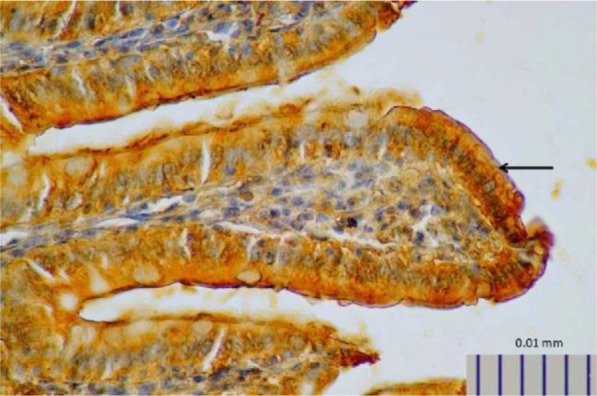
Representative image of intestinal 5-HT in rats treated with *L. plantarum* IS-19596 (400× magnification).

**Fig. 3.**
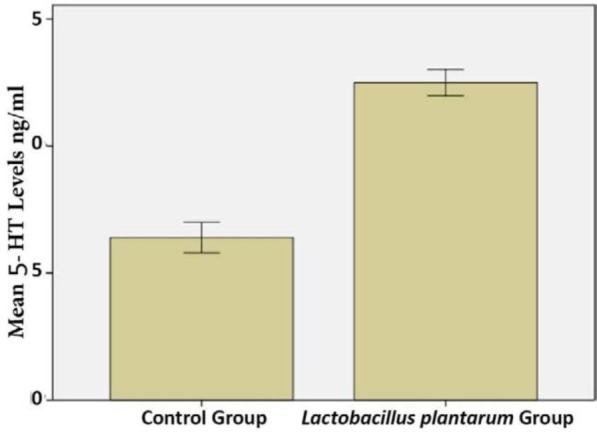
Quantitative analysis of intestinal 5-HT levels in *L. plantarum* IS-10506 treated and control rats. Data represent mean ± SE (n = 10/group).

**Fig. 4.**
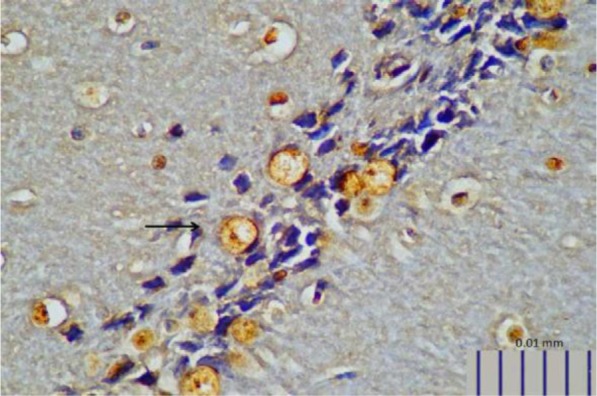
Representative image of brain BDNF expression in control rats, as detected by immunohistochemistry (400× magnification).

**Fig. 5.**
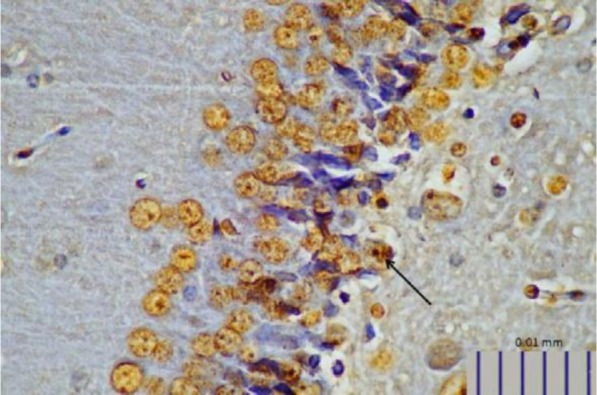
Representative image of brain BDNF expression in rats treated with *L. plantarum* IS-10506 (400× magnification).

**Fig. 6.**
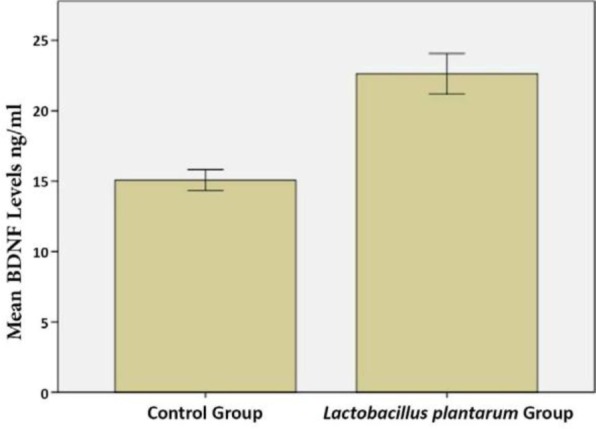
Quantitative analysis of intestinal BDNF levels in *L. plantarum* IS-10506 treated and control rats. Data represent mean ± SE (n = 10/group).

**Fig. 7.**
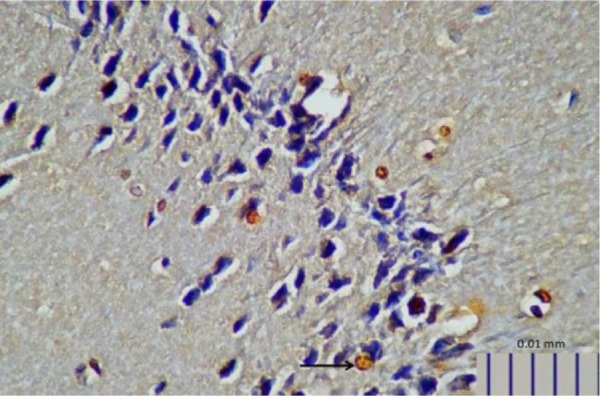
Representative image of brain 5-HTT expression in control rats, as detected by immunohistochemistry (400× magnification).

**Fig. 8.**
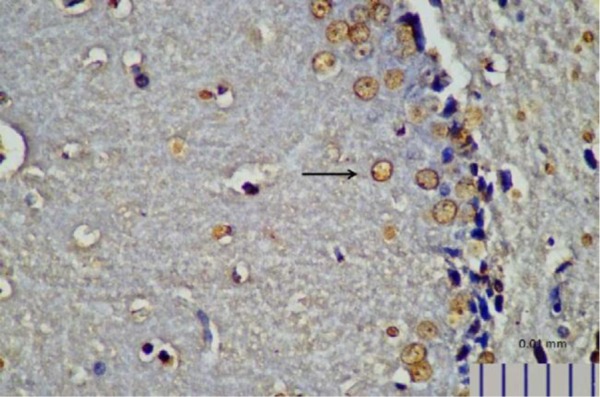
Representative image of brain 5-HTT expression in rats treated with *L. plantarum* IS-10506 (400× magnification).

**Fig. 9.**
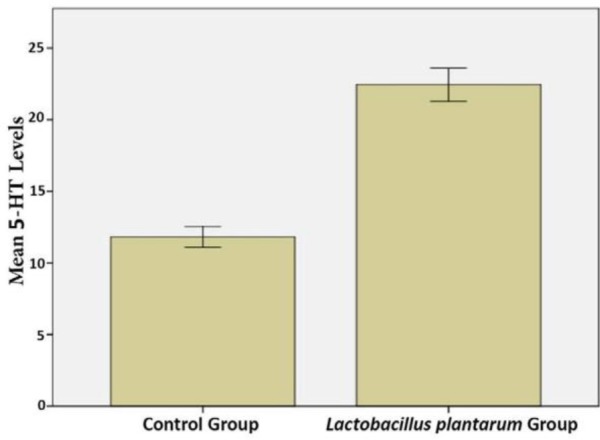
Quantitative analysis of brain 5-HTT levels in *L. plantarum* IS-10506 treated and control rats. Data represent mean ± SE (n = 10/group).

**Fig. 10.**
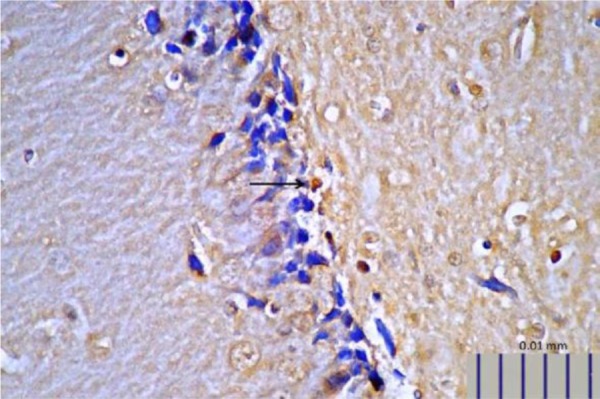
Representative image of brain NT expression in control rats, as detected by immunohistochemistry (400× magnification).

**Fig. 11.**
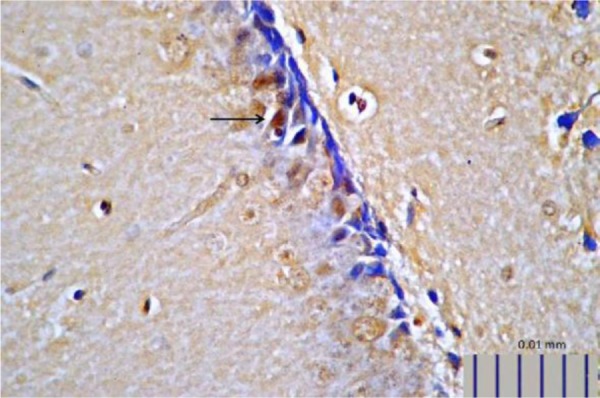
Representative image of brain NT expression in rats treated with *L. plantarum* IS-10506 (400× magnification).

**Fig. 12.**
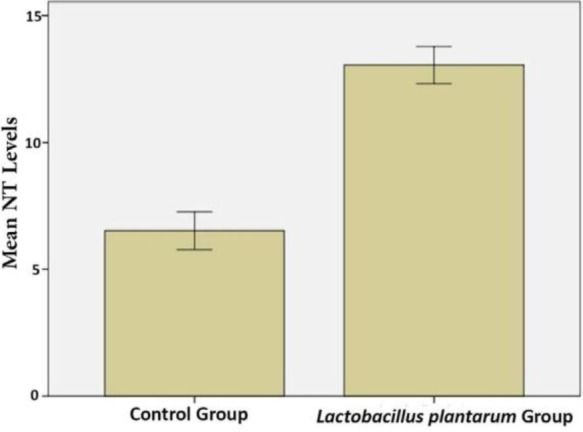
Quantitative analysis of brain NT levels in *L. plantarum* IS-10506 treated and control rats. Data represent mean ± SE (n = 10/group).

## DISCUSSION

A growing body of evidence indicates that gut–brain communication is influenced by the gut microbiome (5, 12, 14). The results of the present study demonstrate that a probiotic bacterium, which is a typical gut microbe, influences neurotransmitter and neurotrophin expression in the brain and intestine.

5-HT is released from enterochromaffin cells of the gastric mucosa in response to various stimuli including signals from gut microbiota (15–17). Although 5-HT is a well-known neurotransmitter that is associated with emotion and behaviour, about 90% of 5-HT in the body is produced in the digestive tract where it is involved in the detection of resource availability and regulation of bowel movement. In platelets, 5-HT contributes to haemostasis and in the central nervous system (CNS), it regulates mood, cognition, appetite, and sleep. 5-HT is also associated with bone metabolism and organ development (8, 9, 17). Gut microbiota stimulates host intestinal cells to produce 5-HT. An imbalance of 5-HT in the brain can lead to depression, whereas peripheral dysregulation of this neurotransmitter has been linked to a variety of diseases (1, 2, 7, 12, 15, 16, 18–21). In this study we found that treatment with *L. plantarum* IS-10506 caused an increase in brain 5-HT level in rats relative to the control group.

BDNF is the most abundant and widely distributed growth factor in the CNS and is important for neuronal survival, migration, and differentiation; axonal and dendritic growth; synapse formation; regulation of synaptic plasticity and behaviour (1, 10, 14, 16, 22); and hippocampal neurogenesis (23). We found here that brain BDNF expression in the hippocampus was enhanced by *L. plantarum* IS-10506 treatment relative to untreated rats, implying that probiotic supplementation can enhance brain development, especially memory and brain plasticity.

5-HTT may contribute to the maintenance of BDNF equilibrium and brain function. Gut microbes are thought to regulate 5-HTT expression (24). Previous studies have shown that the supernatant of *Lactobacillus rhamnosus* GG (LGG) cultures increases 5-HTT expression in mouse intestine (25), whereas *Lactobacillus acidophilus* and *Bifidobacterium longum* culture supernatants have the same effect in intestinal epithelial cells (2). In this study, the number of hippocampal neurons expressing 5-HTT was higher in rats that received *L. plantarum* IS-10506 supplementation than in those without treatment, suggesting that probiotics enhance serotonergic function in the brain.

NTs constitute a family of proteins that promote the survival, development, and function of neurons, in part by inducing BDNF release from hippocampal neurons (26). We observed that *L. plantarum* IS-10506 treatment increased the level of NT in the brain, which corresponded to the elevation in BNDF level. Thus, *L. plantarum* IS-10506 supplementation may enhance neuronal survival, differentiation, and growth.

## CONCLUSION

The results of this study indicate that *L. plantarum* IS-10506 treatment can increase intestinal 5-HT and brain 5-HTT, BDNF, and NT expression in adult rats. These findings suggest that probiotics can promote brain development and function and offer a model for investigating the effects of exogenous factors on the gut–brain axis.
